# A People-Counting and Speed-Estimation System Using Wi-Fi Signals

**DOI:** 10.3390/s21103472

**Published:** 2021-05-16

**Authors:** Liping Tian, Liangqin Chen, Zhimeng Xu, Zhizhang (David) Chen

**Affiliations:** 1 School of Physics and Information Engineering, Fuzhou University, Fuzhou 350000, China; N181110016@fzu.edu.cn (L.T.); chenlq2020@fzu.edu.cn (L.C.); zdchen@fzu.edu.cn (Z.C.); 2 Department of Electrical and Computer Engineering, Dalhousie University, Halifax, NS B3J 1Z1, Canada

**Keywords:** number of persons, walking speed, entering and leaving, channel state information, variance threshold, dynamic time warping, Wi-Fi

## Abstract

Counting the number of people and estimating their walking speeds are essential in crowd control and flow. In this work, we propose a system that uses prevalent Wi-Fi signals to identify the number of people entering and leaving a room through a door. It selects the best subcarrier of Wi-Fi signals and applies the Hampel filter to remove outlier information first. Then, it employs a double threshold method to determine the start and end times of entering or leaving. Afterward, it compares the detected signals with the precollected database using the dynamic time-warping algorithm and determines the number of people. It uses a variance threshold method to identify the states of entering or leaving. It also employs a nonlinear fitting approach to calculate the walking speeds. The experiments show that, in a large empty laboratory, the accuracy rates in determining the number of people are 100% for one person, 81% for two persons, and 95% for three persons. In a small office, the accuracy rates for detecting the number of people are 98% for one or two persons, 82% for three persons, 93% for four, and 75% for five persons. For the walking speed estimation, the accuracy rate for a speed error of less than 0.2410 m/s is 75% for a single person.

## 1. Introduction

Counting people and determining their walking speeds and directions can find applications in many areas. For example, in a smart home, lighting, heating, and cooling can be controlled based on the number of people counted in a room. In a shopping mall, consumption habits and preferences can be analyzed based on the number of people and the time they stay in an area. Public places, such as subways, bus stops, railway stations, passenger flows, and traffic, can be managed and diverted based on crowd densities.

The traditional methods of counting people, including manual counting or infrared imaging, are time consuming, expensive, and sometimes impractical, especially in densely populated areas. Optical image processing with machine learning capability has been introduced and used in various scenarios [1,2,3,4]. However, image-based methods have a few disadvantages, such as performance dependence on optical sensors, potentially large blind areas, and relatively low accuracy in complex environments due to the similarity of multiple objects and the occlusion of targets. Radio-based counting methods then emerged. Some radio methods require people to carry active devices, which emit radio frequency (RF) signals for processing and extracting information [5,6,7]; they can often be inconvenient and impractical. Other radio methods are passive: they do not require users to carry devices, but they need to deploy a wireless sensor network in advance, which can be expensive and challenging with operation overheads.

Fortunately, wireless networks for cellular and internet communications have become widespread and prevalent in recent years. Wi-Fi routers and signals are available in most homes and offices. Wi-Fi signals propagate everywhere and are reflected or scattered by objects and human bodies. Therefore, they carry information about people and their surroundings, and they can be utilized for sensing and detecting human behaviors and activities. For example, they can be used to recognize different human postures and gestures [8,9,10,11,12], to identify people [13,14], to recognize object shapes [15], and to track locations of humans and animals [16,17]. They can also be used to estimate the respiratory rate of a person [18,19,20,21,22] by examining amplitude changes and phase shifts of the channel state information (CSI) of Wi-Fi signals.

In people counting (the topic of this paper), a few methods using the WiFi signals are developed. Seifeldin M et al. proposed the Nuzzer system [23]. The system uses the variance of a received WiFi signal strength to estimate the number of people. Xi et al. proposed the FCC system [24]. The system analyzes the relationship between the number of active people in a region and the channel state information (CSI) it receives. It measures the percentage of non-zero elements in the CSI matrix. Then it uses the gray model theory to relate the percentage with the number of people, obtain its growth curve, and find the number of people [25]. Depatla et al. analyze the absorption loss and multipath resulted from blockage and reflection by a human body [26]. Then they develop a mathematical model that is used to estimate the number of people. Fadel Adib and Dina Katabi [27] apply the principle of inverse synthetic aperture radar and use the multiple input multiple output interference techniques to eliminate the reflected signals of stationary targets. They then propose a method that can identify moving targets and estimate the number of targets. Yang et al. propose a first door-monitoring system by analyzing the WiFi signals [28].

However, all of these methods require training with prior knowledge of training data, which may not always be available and feasible. For this reason, we propose a Wi-Fi signal-based human flow detection method, which requires no data training but a predeveloped sample database. Moreover, Wi-Fi signals can also estimate the walking speeds of a person. The main technical contributions of this paper are summarized as follows:

(a) A double threshold technique is proposed to detect the start and end times of entering or leaving a room. It overcomes the issues of inaccuracy and large computational expenditures that are associated with the conventional manual algorithm described in [29] and the sliding variance method.
(b) An estimation method is developed for the walking speeds of a person with a nonlinear curve-fitting technique.
(c) A detection method is developed to determine the number of people entering or leaving a room. Unlike the deep learning algorithm, such as the door monitoring method presented in [28], this method requires no data training, but a predeveloped sample database.
(d) A variance method is proposed to determine the states of entering or leaving. It utilizes the differences between the signal variances inside and outside a room—a feature that has not been explored or reported in the literature so far. It leads to a relatively simple computation algorithm that requires only one receiving antenna (unlike the two-antenna approach presented in [28]).

## 2. Materials and Methods

**Figure 1:**
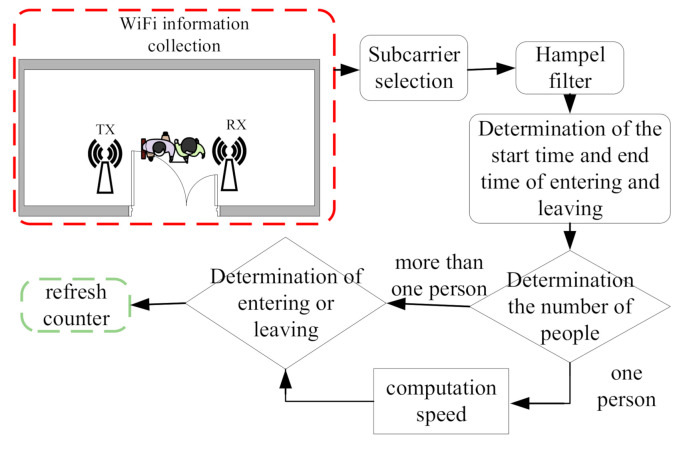
The overall flowchart of the proposed algorithm.

Figure 1 is the overall flowchart of the proposed method. The scenario under consideration is a room with a door, and people enter or leave the room through the door. Wireless routers are inside the room and placed close to the door. The subcarriers from the Wi-Fi router are collected, and one of them is selected for further processing. A filter is used to remove the outliers of the selected subcarrier. A threshold method is then applied to detect the starting and end times of entering or leaving a room through the door. The dynamic time warping (DTW) algorithm is used to compare and analyze the detected signals with a preconstructed database. The number of people going through the door is then determined. After this, a variance method is employed to decide whether a person is entering or leaving. If it is a single person, the speed is also being estimated. 

The mainstream Wi-Fi system uses 802.11 a/g/n that employs orthogonal frequency division multiplexing (OFDM). It divides the bandwidth of 20 MHz into 56 subcarrier bands. These subcarriers carry the channel state information (CSI) in their amplitudes and phases. When encountering an object, these carriers experience different frequency-dependent scattering, which leads to signal multipath propagation and fading; they result in the changes in the received CSI amplitudes and phases that are associated with the object. The changed information can be used and processed for the detection of the object. In other words, the Wi-Fi routers and signals commonly available in a room are utilized for the sensing and counting of people entering and leaving through a door. In the following subsections, we elaborate on the proposed system.

### 2.1. Selection and Filtering of the Subcarriers 

**Figure 2:**
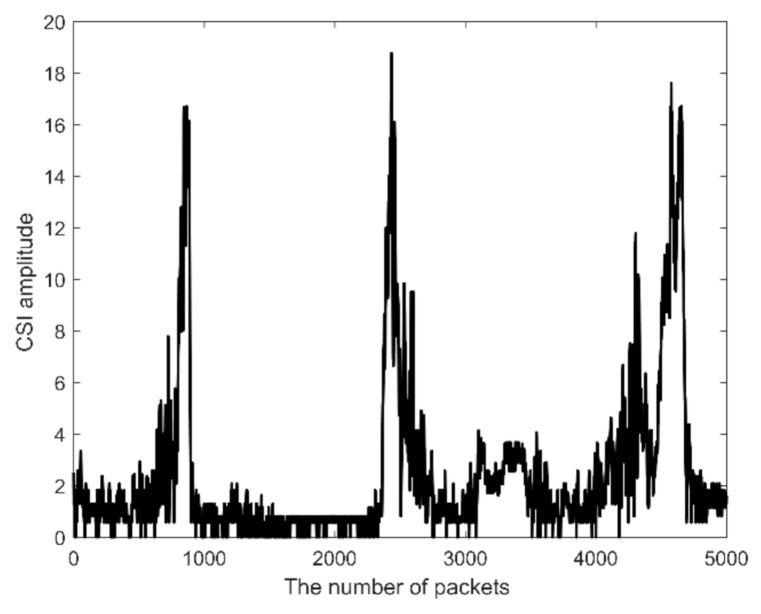
The signal before the filtering.

In the proposed algorithm, we select the subcarrier with the largest variance for detection processing. Because it has a largest variance, the chosen subcarrier is more sensitive to the changes in the CSI than other subcarriers. In the scenario we consider, the frequency response of a subcarrier can be expressed as follows:

(1) $${\mathrm{CSI}}_{i}=\sum_{k=0}^{K}r_{k}e^{-j2\pi f_{i}\tau_{k}}$$

where *K* is the number of signal multipath, *r_k_* is the signal path amplitude through path *k*, *f_i_* is the frequency of the subcarrier with the largest variance, and *τ_k_* is the signal travel time through path *k*. Figure 2 shows an example of the subcarrier that has the largest variance, and it is the 15th subcarrier. 

**Figure 3:**
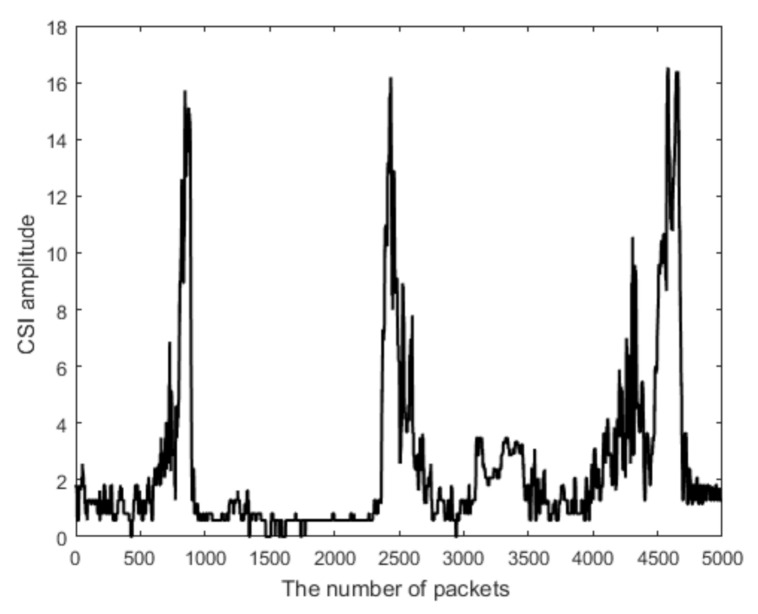
The signal after the Hampel filtering.

The signal of the 15th subcarrier is then sent to the Hampel filter [30] to remove the outliers, which have significantly different values from the other neighboring CSI measurements. Although the CSI signal is not normally distributed for a long period, it is approximately distributed within a short time. Therefore, we use the Hampel filter. We tried other filters, such as the median filter; they do not work as well as the Hampel.This comparison indirectly verifies the appropriateness of the use of the Hampel filter. Figure 3 shows the normalized signal after the filter, and the outlier is removed. In comparison to the signal of Figure 2 before the filtering, the signal of Figure 3 is much cleaner. 

### 2.2. Determination of the Start and End Times 

**Figure 4:**
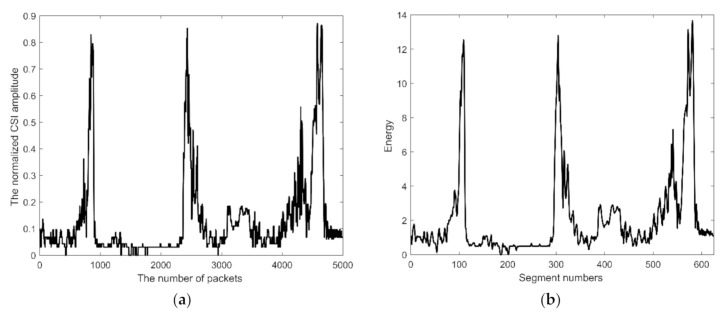
(**a**) The normalized signal. (**b**) The short-time energy of the normalized signal.

In order to reduce the influence of the environment, the CSI signal is normalized before we determine the start and end times. Figure 4a shows the result of normalization. The signal after the filter, *x*(*n*), is further windowed and segmented with the function *w*(*n*). It becomes *y_i_*(*n*):

(2) $$y_{i}(n)=w(n)\cdot x[(i-1)\cdot n_{s}+n],\;1\leq n\leq L,\;1\leq i\leq f_{n},$$

where *y_i_*(*n*) represents the segmented and windowed signal, *x_i_*(*n*) represents the signal after the filter, *w*(*n*) represents the window function, *n_s_* represents the segment length, *n* represents the segment shift number, *L* represents the total number of segments, and *f_n_* represents the number of segments in each packet.

The short-term energy expression of each segment is calculated with the following equation:

(3) $$E(i)=\sum_{n=0}^{L-1}y_{i}^{2}(n),1\leq i\leq f_{n}$$

where *E*(*i*) represents the energy of the signal. Figure 4b shows the short-term energy of the segmented signal. 

The following double threshold method is proposed to detect the start and end times of entering or leaving the room. Assume that the wireless routers are inside, by the door: entering is considered walking from outside the door, past the routers, and into the room; leaving is considered walking from inside the room, past the routers, and out of the room. The Wi-Fi signals inside a room are more concentrated and sensitive to environmental changes (including human walking) than those out of the room and outside the room. 

When a person goes past the door, the received Wi-Fi signal will experience a large disturbance. Two threshold values are then preselected to determine the start and end times of entering or leaving: a high threshold ***amp1*** corresponds to a person’s passing by the routers and initiates the crowd counting process. A smaller threshold ***amp2*** determines the start and the end time of the entering or leaving. In other words, once the signal reaches above ***amp1***, the proposed method searches to both the left and right sides of ***amp1*** in time to find the time instances at which the signal strengths are equal to ***amp2***. The time instant on the left side of ***amp1*** (or before the time of ***amp1***) that equals ***amp2*** is the start time. The time instant on the right side of ***amp1*** (or after the time of ***amp1***) that equals ***amp2*** is the end time. Moreover, the minimum time of entering and leaving is preselected. If the time difference between the start and the end time is less than the minimum time, the signal detected is considered an interference signal and ignored. Initially, we take ***amp1*** to be equal to one-half of the maximum value of amp and ***amp2*** to be equal to one-eighth of the maximum value of amp. They are then adjusted manually during the process of developing the database. The computational algorithm for the double threshold method is shown below (Algorithm 1). 

**Figure 5:**
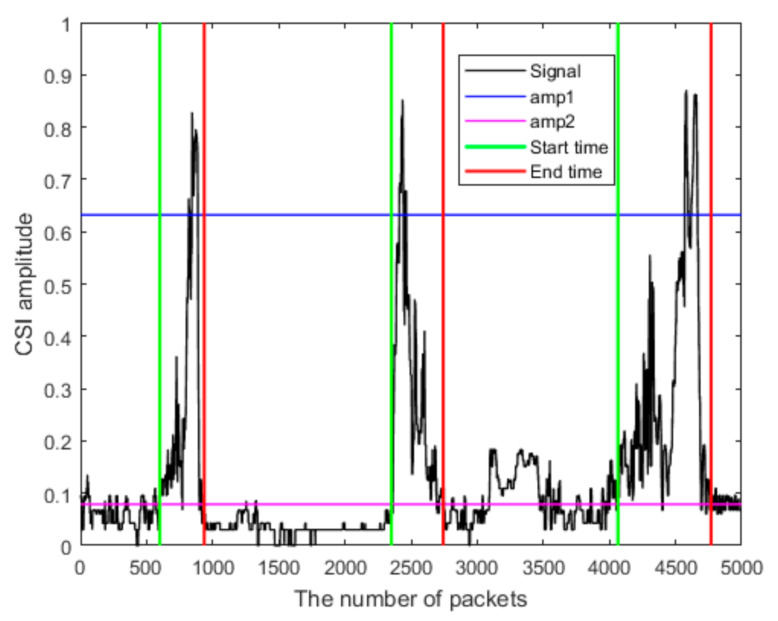
Determination of the start and end times of entering or leaving.

Figure 5 shows the result of applying the double threshold method that leads to determining the start and end times for entering or leaving a room. Note that the normalization is applied to the short-term energy to remove the adverse effects of the relative differences among different data packets.
**Algorithm 1:** Determination of the start and end times.  Input: amp, amp1, amp2,   Output: v_Begin, v_End   for *n* = 1: length(amp)     switch status     case {0, 1}       If amp(*n*) > amp1       Identify the entering and leaving stage;       else if amp(*n*) > amp2       May be the entering and leaving stage;   else           No one entering and leaving;        End if   case 2        if amp(*n*) > amp2      Keep entering and leaving stage;        else      entering and leaving stage will end;        else          End of entering and leaving stage;         End if   case 3        Record the current stage and look for the next stage     End switch   end for

### 2.3. Determination of the Number of People

Once the start and end times are determined, the signals detected between the start and end times are processed to find the number of people. The processing is conducted in comparison with a signal database, which has been developed in advance. The database is assumed to have been developed for eight scenarios: one person entering, one person leaving, two persons entering, two persons leaving, three persons entering, three people leaving, one person entering and leaving with a child, and persons carrying a child.

The similarity between the detected signals and the database is measured and used to determine the number of people. However, the length of the signal detected and analyzed can be different from that of the database since it changes with time. If a simple reduction or extension of signal length is applied, the results are not accurate. To address this issue, we adapt the dynamic time warping (DTW) algorithm. The DTW algorithm is proposed in [31], and it is used to solve the problem of uneven speaking speeds in the speech recognition of isolated words. Here, we adapt the DWT for measuring similarity between the length-varying signal collected and the database.

More specifically, the DTW is an optimization algorithm. In application to our case, it is used to find the minimum Euclidean distance between the signal data received and the sample data in the database:

(4) $$D=\min\sum_{i=1}^{L}d[x(i),R_{k}(i)]\;\begin{array}{c} k=1,2,\ldots ,N, \end{array}$$

where *x*(*t*) represents the signal (the signal between start time and end time), $R_{k}(i)$ represents the *k*th data set of the database, and *L* represents the total number of the segments in the database. *N* is the number of the datasets. Finding the number of persons is to search for the number of people that give the minimum *Euclidean* distance or smallest *D* of (4).

### 2.4. Determination of the State of Entering and Leaving 

**Figure 6:**
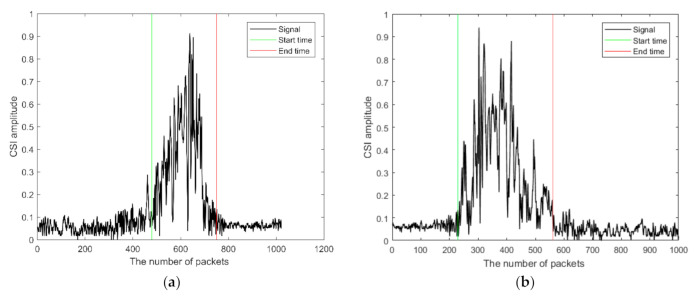
The interval between entering and leaving. (**a**) Leaving the room. (**b**) Entering the room.

Once the number of people is determined, their state of entering or leaving needs to be decided. A variance threshold method is developed to carry out the task. The CSI signal strength received is shown in Figure 6a when a person is leaving and in Figure 6b when a person is entering. Since the Wi-Fi routers are placed inside a room, the signal inside is more cluttered than outside, mainly due to the signal multipath. Therefore, the signal variance inside is larger than that outside. As a result, the variance threshold method shown below is proposed to determine the state of entering or leaving. More specifically, the following formula is used for judging the state of entering or leaving:

(5) $$\left\{\begin{array}{c} \mathrm{If}\;v_{t-1}\geq v_{T}\&v_{t+1}<v_{T}\Rightarrow \mathrm{entering}\\ \mathrm{If}\;v_{t-1}\leq v_{T}\&v_{t+1}>v_{T}\Rightarrow \mathrm{leaving} \end{array}\right.$$

where *v_t−_*_1_ represents the variance of the interval signal in the previous segment and *v_t+_*_1_ represents the variance of the gap signal in the next period. *v_T_* represents the threshold that is the mean value of *v_t−_*_1_ and *v_t+_*_1_.

### 2.5. Determination of the Velocity 

The walking speed of a person has an impact on the number of data packets received. 

**Figure 7:**
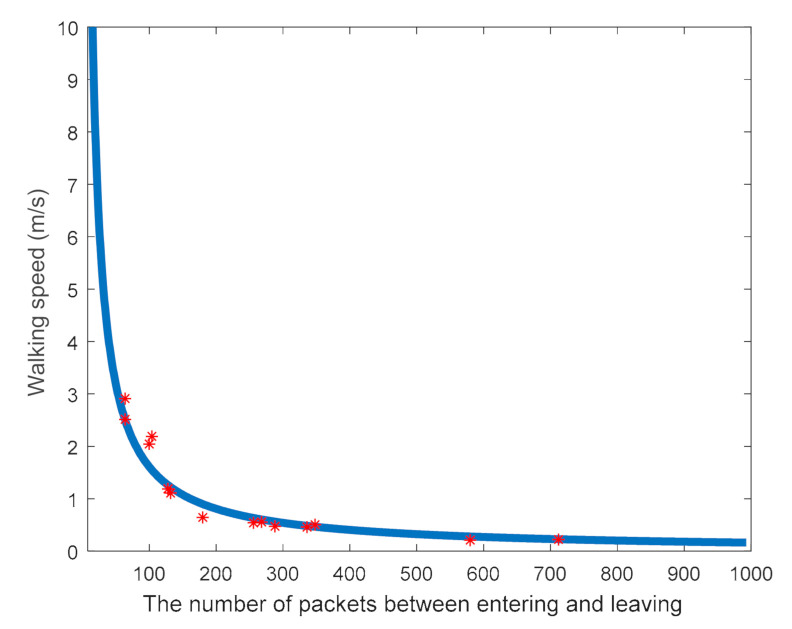
The curve-fitting relationship of velocity versus the number of data packets received.

Their relationship is measured during the development of the database. The result is shown in Figure 7 in our case. In this paper, the method of curve fitting of a rational function is used. The fitting algorithm took 0.33 s in this paper. The following is the curve-fitting equation for the speed estimation:

(6) $$u=\frac{p_{1}}{L+p_{2}},\;p_{1}=162.7,\;p_{2}=-6.01$$

where *u* represents the speed of entering or leaving, *p*_1_ and *p*_2_ are the fitting constants, and *L* represents the number of data packets received between the start and end times. The MATLAB toolkit is used linear fitting. Once (6) is obtained, it is used to determine the walking speed. Then, (6) is used to determine the walking speed. 

## 3. Results

We conducted the experiments using the above method. They are elaborated as follows. 

We used the Wi-Fi transmitter of a desktop equipped with an Intel 5300 NIC. It had one antenna and broadcasts packets into the air. We used the receiver of a desktop equipped with an Intel 5300 NIC. It had three antennas, which formed a uniform linear array. We used a Linux 802.11n CSI Tool [32] to collect the CSI measurements. We employed channel 13 at 2.4 GHz. The transmission rate of packets was set to 100 Hz. We used MATLAB to process the CSI data.

**Figure 8:**
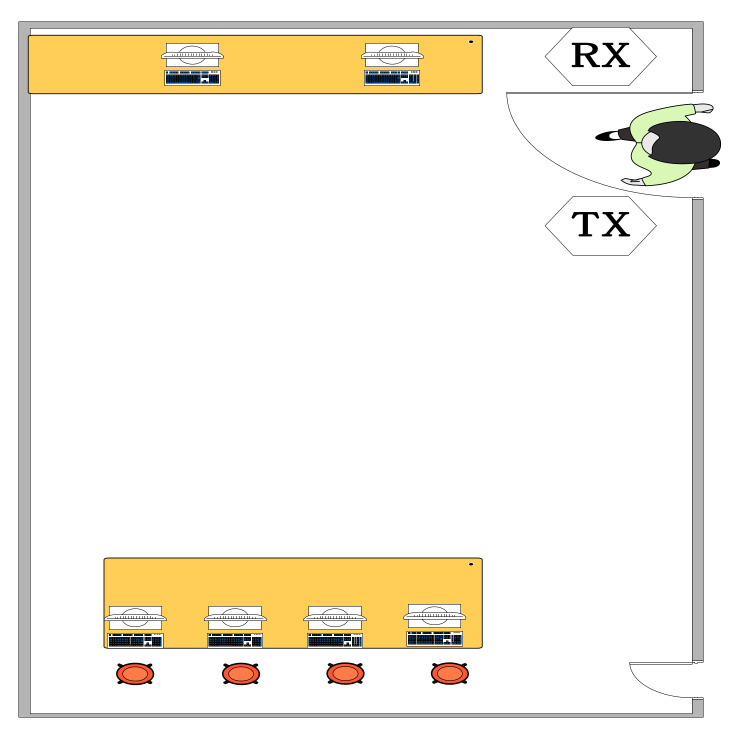
Experiment with a large empty laboratory.

**Figure 9:**
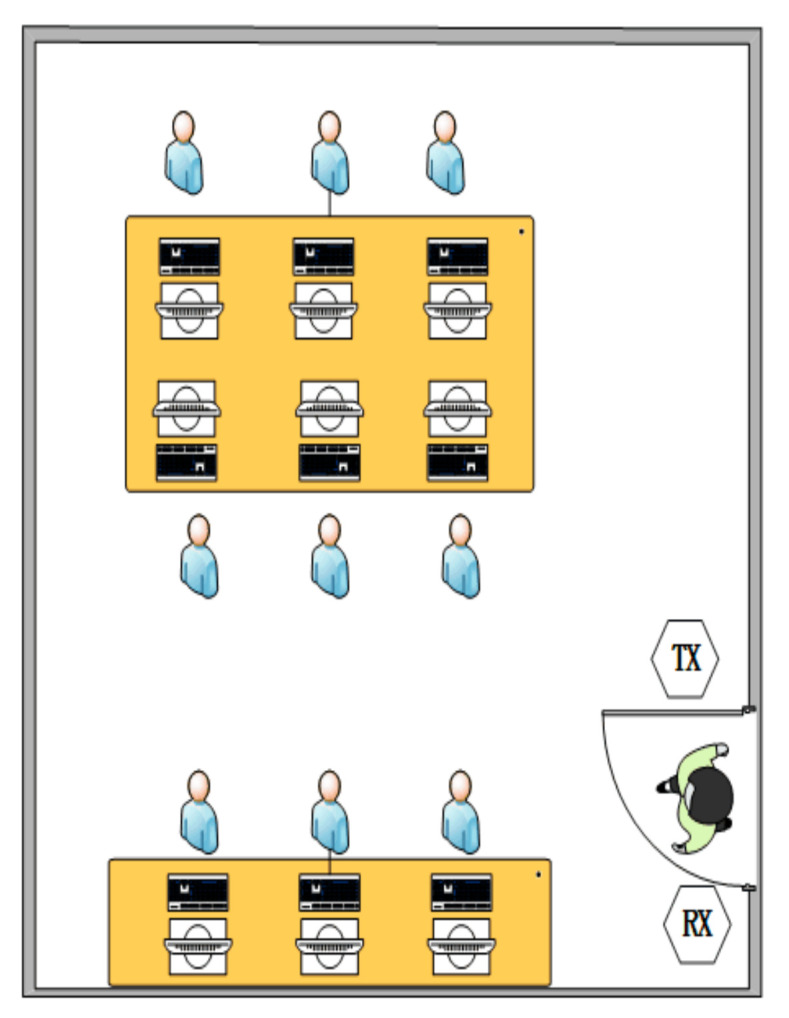
Experimental setup with a small office room.

**Figure 10:**
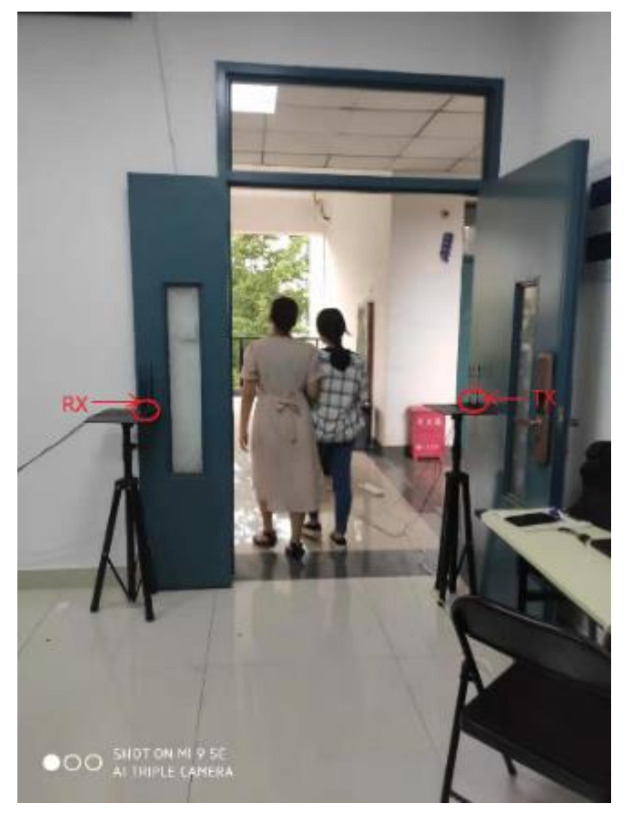
Participants walking through the door.

We conducted the experiments in the two indoor environments: a large empty laboratory and a small office room with furniture and students, as shown in Figure 8 and Figure 9. The transmitter and receiver were placed on both sides of the door at a distance of about 1.2 m apart. There are many desks, chairs, and equipment in the laboratory. Seven volunteers (three males and four females) participated in the experiment. They walked through the door, as shown in Figure 10.

### 3.1. Detection of Passing Directions: Entering or Leaving (Exiting)

**Figure 11:**
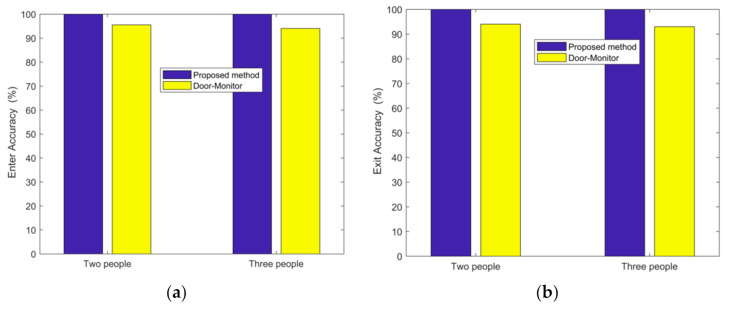
Accuracy of passing direction detection at a large empty laboratory. (**a**) Enter. (**b**) Exit.

In the case of a large empty laboratory, we tested for two persons entering and leaving 100 times, and three persons 103 times. The accuracy of entering is 100% for two persons and 100% for three persons. The accuracy of exiting is 100% for two persons and 100% for three persons. For comparison purposes, the Door-Monitor’s [28] accuracy is also shown in Figure 11. The signal outside the door is weak due to the attenuation by the walls. Walking by humans causes a smaller CSI signal variance than that inside the door. By utilizing the difference, the proposed algorithm is robust. Unlike the method of Reference [28] that uses the two (or more) antennas and the phase difference between them, the proposed method is independent of the number of antennas and can work with a single antenna. 

**Figure 12:**
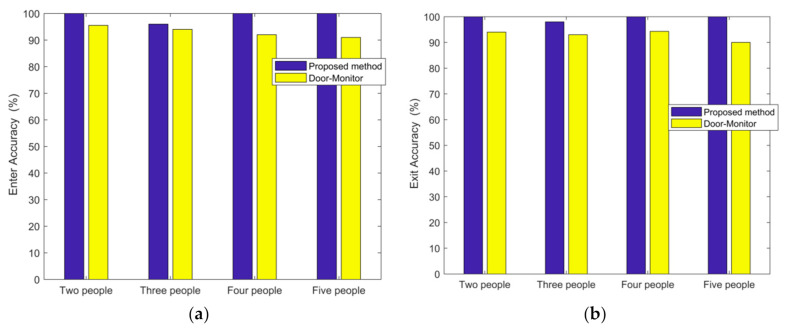
Accuracy of passing direction detection at small office room. (**a**) Enter. (**b**) Exit.

In the case of a small office room with furniture and students, we tested for two persons entering and leaving 103 times, three persons 100 times, four persons 103 times, and five persons 102 times. The accuracy of entering is 100% for two persons, 96% for three persons, 100% for four persons, and 100% for five persons. The accuracy of exiting is 100% for two persons, 98% for three persons, 100% for four persons, and 100% for five persons. For comparison purposes, the Door-Monitor’s [28] accuracy is also shown in Figure 12. 

### 3.2. Determination of the Number of People

In the case of the large empty laboratory, we tested for one person entering or leaving 100 times, two persons 100 times, and three persons 103 times. The accuracy is 100% for one person, 81% for two persons, and 95% for three persons.

Table 1 presents the test results for the number of people in the large empty laboratory. For instance, the fourth column shows that, for the 103 tests of the three-person cases, the result is five tests showing one person (incorrect) and 98 tests showing three persons (correct).

In the case of the small office, we tested the cases of one person 101 times, two persons 103 times, three persons 100 times, four persons 103 times, and five persons 100 times. The accuracy is 98% for one person, 98% for two persons, 82% for three persons,93.2% for four persons, and 75% for five persons.

Table 2 presents the test results for the number of people in the small office. For instance, the fifth column shows that, for the 100 tests of the three-person cases, the result is 10 tests showing two persons (incorrect), three tests showing one person (incorrect), five tests showing four persons (incorrect), and 82 tests showing three persons (correct).

The above test results are applicable for either people entering or leaving. The proposed algorithm can decide the passing directions (whether it is entering or leaving) and then the number of people. It can account for the situation where the number of people entering is different from the number of people leaving. The results of the determination of the number of people entering or leaving are almost identical since the detection of the passing directions is virtually 100% accurate. 

### 3.3. Estimation of the Walking Speed

**Figure 13:**
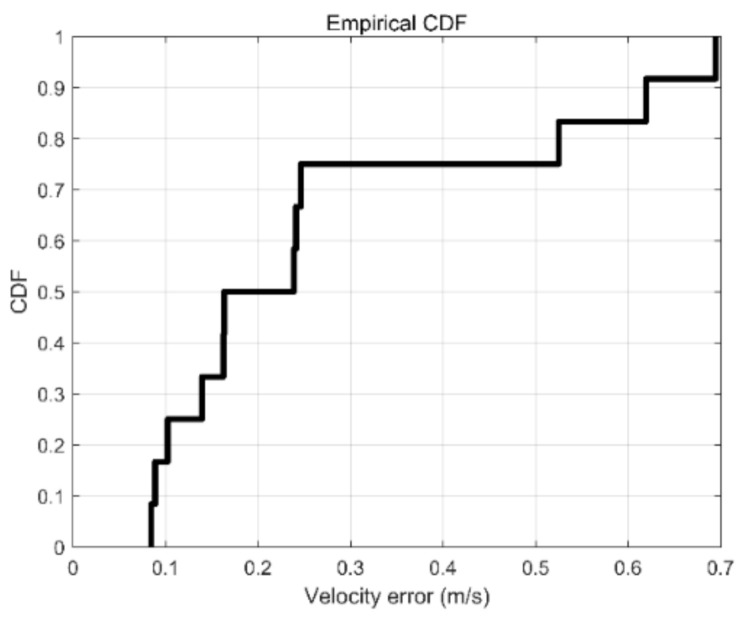
The cumulative density function of the speed detection accuracy.

We tested the case of one person entering and leaving the room 26 times. The cumulative density function for the speed estimation accuracy is shown in Figure 13. As can be seen, the accuracy rate for the speed error of less than 0.241 m/s is 75%. For the speed error over 0.7 m/s, the accuracy rate is 100%.

### 3.4. Impact of the Sizes of the Objects Carried

**Figure 14:**
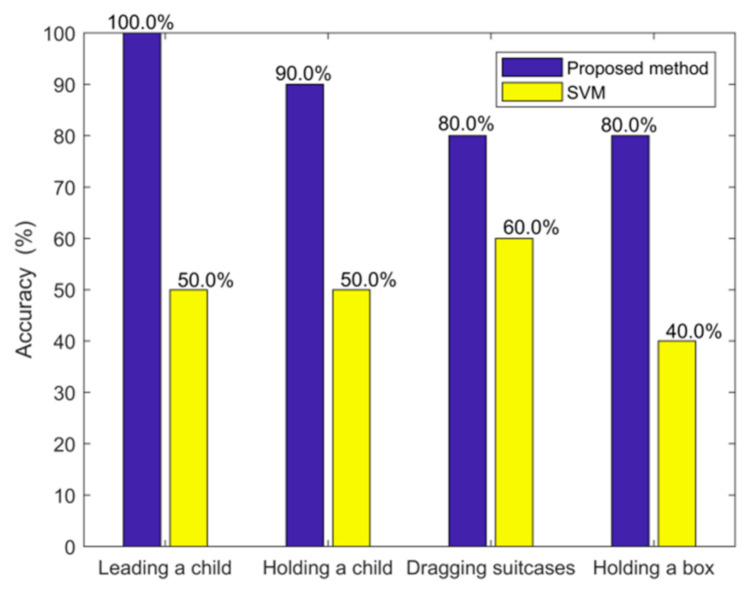
Accuracy in determining the number of persons entering or leaving the room with a child or objects carried.

In many situations, persons entering or leaving a room may be carrying children or objects. Therefore, it is useful to investigate the impact of children and objects on the detection accuracy. Along this line, we tested the following cases: an adult walking with a child, holding a child, dragging a suitcase of size 40 cm × 26 cm × 50 cm, and carrying a carton box on their shoulders. We conducted 10 tests for each case. The results are shown in Figure 14. Our proposed method performs better than the conventional SVM in these scenarios. Note that the SVM has the previous filtering steps and is given the same conditions as those for the proposed method. For example, the SVM approach uses the same predeveloped database. In such a way, we can compare the proposed method and the SVM approach fairly. 

From Figure 14, we can see that dragging an object (either a person or an object) has a higher accuracy than holding an object. This is perhaps due to the spatial separation between the person and the object, and the object being dragged is larger than the object being held. 

### 3.5. Impact of the Disturbance

**Figure 15:**
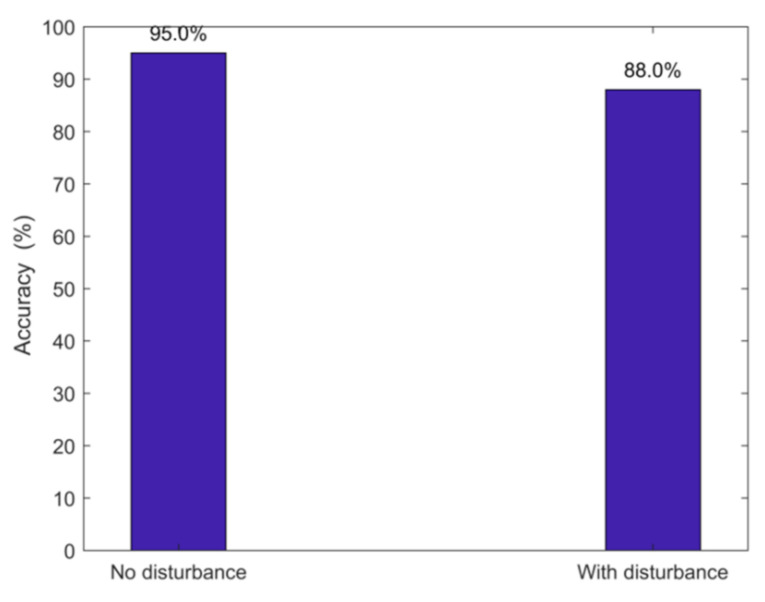
Movement of other people as disturbance.

In order to verify the stability of the algorithm, we considered the influence of people walking indoors on the accuracy of number recognition. The result was shown in Figure 15. If the room is undisturbed, the average identification rate is 95%. The recognition rate is 88% when someone else is moving in the room.

## 4. Conclusions

Counting the number of people entering and leaving a room provides important information in human traffic control and flow analysis. Few papers used CSI signals to estimate the number of people entering and leaving a room. We found only one relevant paper that has been published so far [28]. It uses a deep learning approach and requires training with a lot of data. Our method does not need training. It only requires the predeveloped sample database. In addition, at least two receiving antennas are used in [28] with higher cost and complexity. The proposed method, however, only needs one receiving antenna. It also computes the walking speed. 

The experiments show that, in a large empty laboratory, the accuracy rates in determining the number of people are 100% for one person, 81% for two persons, and 95% for three persons. In a small office, the accuracy rates for detecting the number of people are 98% for one or two persons, 82% for three persons, 93% for four persons, and 75% for five persons. For the walking speed estimation, the accuracy rate for the speed error of less than 0.2410 m/s is 75% for a single person. A group of five people may be considered as a reasonably extreme case due to the size of the door. If more than five people enter or leave the door and they are close to each other, the proposed method will present the result of five people. If they are not close to each other, the proposed method will count them separately.

The proposed algorithm is at the laboratory research stage and is not ready for real use yet. However, the ultimate goal is to make it ready for real use and to embed it in the Wi-Fi router—this paper is the first step development and verification of an algorithm. Its real-time implementation and counting multiple persons entering and leaving simultaneously are topics of future research.

## Figures and Tables

**Table 1:** Test results of the numbers of persons in the case of a large laboratory.

	Actual	One Person (100 Tests)	Two Persons (100 Tests)	Three Persons (103 Tests)
Detected				
One person		100	19	5
Two persons		0	81	0
Three persons		0	0	98

**Table 2:** The test results for the number of persons in the case of a small office.

	Actual	One Person	Two Persons	Three Persons	Four Persons	Five Persons
Detected						
One person		99	0	3	0	0
Two persons		0	101	10	7	0
Three persons		2	0	82	0	0
Four persons		0	2	5	96	25
Five persons		0	0	0	0	75

## Data Availability

Not applicable.
